# Investigating the role of signal transducer and activator of transcription 3 in feline injection site sarcoma

**DOI:** 10.1186/s12917-022-03352-y

**Published:** 2022-07-14

**Authors:** Cheng-Hsin Shih, Yen-Chen Chang, Yun-Chiang Lai, Hue-Ying Chiou

**Affiliations:** 1 Graduate Institute of Molecular and Comparative Pathobiology, School of Veterinary Medicine, National Taiwan University, 10617 Taipei, Taiwan; 2 Graduate Institute of Veterinary Pathobiology, College of Veterinary Medicine, National Chung Hsing University, 402 Taichung, Taiwan

**Keywords:** Feline injection site sarcoma, Chronic inflammation, STAT3

## Abstract

**Background:**

Feline injection-site sarcomas (FISSs) are malignant mesenchymal tumors of different histotypes. The pathogenesis of FISS has been correlated with chronic inflammation, resulting in neoplastic transformation. Activation of the Janus kinase-signal transducer and activator of transcription 3 (STAT3) have been demonstrated to play a critical role in tumor development by regulating signaling pathways involved in cell proliferation, survival, metastasis, and angiogenesis in human medicine. To characterize the role of STAT3 in FISS, we first detected STAT3 and phosphorylated STAT3 in formalin-fixed and paraffin-embedded (FFPE) FISS tissues using immunohistochemical staining.

**Results:**

STAT3 was detected in 88.9% (40/45) of FISS cases, and phosphorylated STAT3 was detected in 53.3% (24/45) of cases. However, the expression levels of both forms of STAT3 were not correlated with tumor grade. To study the role of STAT3 in tumor survival, two primary cells derived from FISSs of two cats exhibiting consistent immunophenotypes with their parental FFPE tissues were established. A dose-dependent inhibitory effect on cell proliferation was observed in both primary FISS cells treated with the STAT3 inhibitor, 5-hydroxy-9,10-dioxo-9,10-dihydroanthracene-1-sulfonamide.

**Conclusions:**

The STAT 3 may play an important role in the tumorigenesis of FISS and be a potential molecular therapeutic target for FISS.

**Supplementary Information:**

The online version contains supplementary material available at 10.1186/s12917-022-03352-y.

## Background

Feline injection-site sarcoma (FISS) has been recognized worldwide for over 20 years. It was previously called vaccine-associated sarcoma and was first described in 1991 [1, 2]. It was first proposed that the majority of fibrosarcomas arose in vaccination sites. Histologically, these neoplasms were surrounded by characteristic inflammatory infiltrates of lymphocytes and macrophages containing foreign materials identical to those previously described in post-vaccinal inflammatory injection site reactions. By electron probe X-ray microanalysis, aluminum oxide, which is an adjuvant in many feline vaccines, was identified in the surrounding macrophages [3–8]. The term “vaccine-associated sarcoma” is no longer appropriate because, in addition to vaccines, injection of foreign materials, such as long-acting antibiotics, steroids [6], lufenuron [9], meloxicam [10], cisplatin [11], or even microchip [12] in the subcutis or muscle of a predisposed cat can induce a chronic inflammatory response and ultimately neoplastic transformation. Following the observations that the development of FISSs was not necessarily due to vaccination, the new term “injection site sarcomas” was considered more compatible with the condition and has since gained wide acceptance.

The etiology of FISSs remains obscure; however, the hypothesis of FISSs, which may arise at the injection site as a result of chronic inflammation, is generally accepted. Chronic inflammation is considered to be inflammation of prolonged duration and is a sequela of acute inflammation if there is a failure to eliminate the agent or substance that incites the process. It is characterized by infiltration of lymphocytes, plasma cells, and macrophages, accompanied by tissue necrosis and tissue repair, such as wound healing, fibrosis, and granulation formation [13]. The potential role of chronic inflammation in the development of FISSs has already been discussed in the past decade owing to its histopathological features of the presence of an infiltrate of lymphocytes and macrophages at the tumor periphery, and is reminiscent of a persistent inflammatory response. Additionally, FISSs are primarily composed of the proliferation of spindle cells immunohistochemically and ultrastructurally as fibroblasts and myofibroblasts, which play a major role in chronic inflammation and wound healing [8, 14]. Cellular factors that promote inflammation, such as acidic fibroblast growth factor (FGF-a) and basic FGF (FGF-b), create a favorable environment for the expression of oncogenes and subsequent development of tumors [14]. Expression of p53, FGF-b, and transforming growth factor-α have been reported in FISSs, indicating the participation of these proliferation factors in the pathogenesis [14]. FISSs are histologically similar to feline ocular post-traumatic sarcomas, suggesting a common pathogenesis of inflammation and wound healing in the development of these mesenchymal tumors [15].

The connection between tumorigenesis and inflammation is mediated via intrinsic and extrinsic pathways [16]. The extrinsic pathway is driven by inflammatory or infectious conditions that increase the risk of cancer, and the intrinsic pathway is driven by genetic alterations such as oncogene activation and/or inactivation of tumor-suppressor genes that cause inflammation and neoplasia. Both pathways interfere with tumor cells and induce the activation of several key transcription factors in tumor cells leading to tumor-associated inflammation, including nuclear factor-κB (NF-κB), signal transducer and activator of transcription 3 (STAT3), and hypoxia-inducible factor 1 α, impacting any stage of tumorigenesis, such as initiation, promotion, progression, and metastasis [17]. These transcription factors coordinate the production of inflammatory mediators, including chemokines, cytokines (mainly interleukin [IL]-1, tumor necrosis factor, and IL-6), prostaglandins, and cyclooxygenase 2 production by tumor cells. Cytokines activate the same key transcription factors in inflammatory, stromal, and tumor cells, resulting in the production of more inflammatory mediators and a cancer-related inflammatory microenvironment that triggers tumor growth and invasiveness [16, 17].

Among the transcription factors aforementioned, STAT3 and NF-κB are critical for tumor-related inflammation. A positive association between NF-κB activation and tumor-associated inflammation is evidenced by colitis-associated colon cancer and hepatitis-associated hepatocellular carcinoma in murine models [18, 19]. In our previous study, NF-κB expression was confirmed in FISS, and there were dose-dependent inhibitory effects on the growth of FISS primary cells treated with an NF-κB inhibitor [20]. Similar to NF-κB, STAT3 is a point of convergence for numerous oncogenic signaling pathways. Transient STAT3 activation is a critical determinant for the restoration of tissue integrity [21], wound healing, and ultimately the resolution of the immune response. STAT3 plays a key role in normal cell growth, such as embryogenesis, and is constitutively activated in approximately 70% of solid and hematological cancers in human medicine, and is involved in cell proliferation, survival, angiogenesis, and tumor-mediated immune evasion [22]. Augmented tumor cell-intrinsic STAT3 signaling activity promotes tumor progression as well as tumor-extrinsic immunosuppressive effects. It modulates the surrounding stromal and variable types of immune cells by stimulating the production of pro-inflammatory cytokines, suppressing the secretion of type I interferons and associated interferon response genes, and supporting immunosuppression [23]. STAT3-dependent feedforward signaling loops are formed, which further fuel tumor progression. In normal cells or under physiological conditions, the activation of STATs is rapid and transient because they are negatively regulated by several proteins such as suppressors of cytokine signaling and protein inhibitor of activated STAT [24, 25]. Conversely, aberrant and persistent activation of STAT3 was observed in both tumor cells and adjacent stromal and immune cells.

In veterinary medicine, STAT3 expression has been reported in canine, feline, and equine mammary gland tumors [26, 27], equine renal hemangiosarcoma [28], feline oral squamous cell carcinoma [29], canine diffuse large B-cell lymphoma [30], and feline injection-site sarcomas [31]. In a previous veterinary study, the nuclear positive rate of phospho-STAT3 (Tyr705) was 68.89% (31/45) in feline mammary gland tumors [32] and 48.1% (13/27) in feline oral squamous cell carcinoma (OSCC) [29]. In previous human studies, nuclear phospho-STAT3 (Tyr705) was immunoreactive in 37–67% of head and neck squamous cell carcinoma samples and 49.3% of hepatocellular carcinomas (HCC) [33]. Considering that FISS is regarded as a chronic inflammation-associated tumor with histological features that are similar to wound healing, the expression of STAT3 may be significant and plays an important role in tumorigenesis.

STAT3 is highly expressed in FISS [31]. However, the expression level of phosphorylated STAT3, which is a prognostic factor in many human cancers [33–35], remains unknown in FISSs. This study aimed to characterize the expression of STAT3 and phosphorylated STAT3 (Tyr705) in FISSs and their derived primary cells and to investigate the role of STAT3 in FISS cellular survival using the STAT3 inhibitor LLL12. This study could enhance our knowledge of the sarcomagenesis of FISSs and might also identify potential therapeutic targets for FISS.

## Results

### Detection of STAT3 and phospho-STAT3 (Tyr705) expression and their correlation with histopathological grading in FISS cells by immunohistochemistry (IHC)

**Fig. 1:**
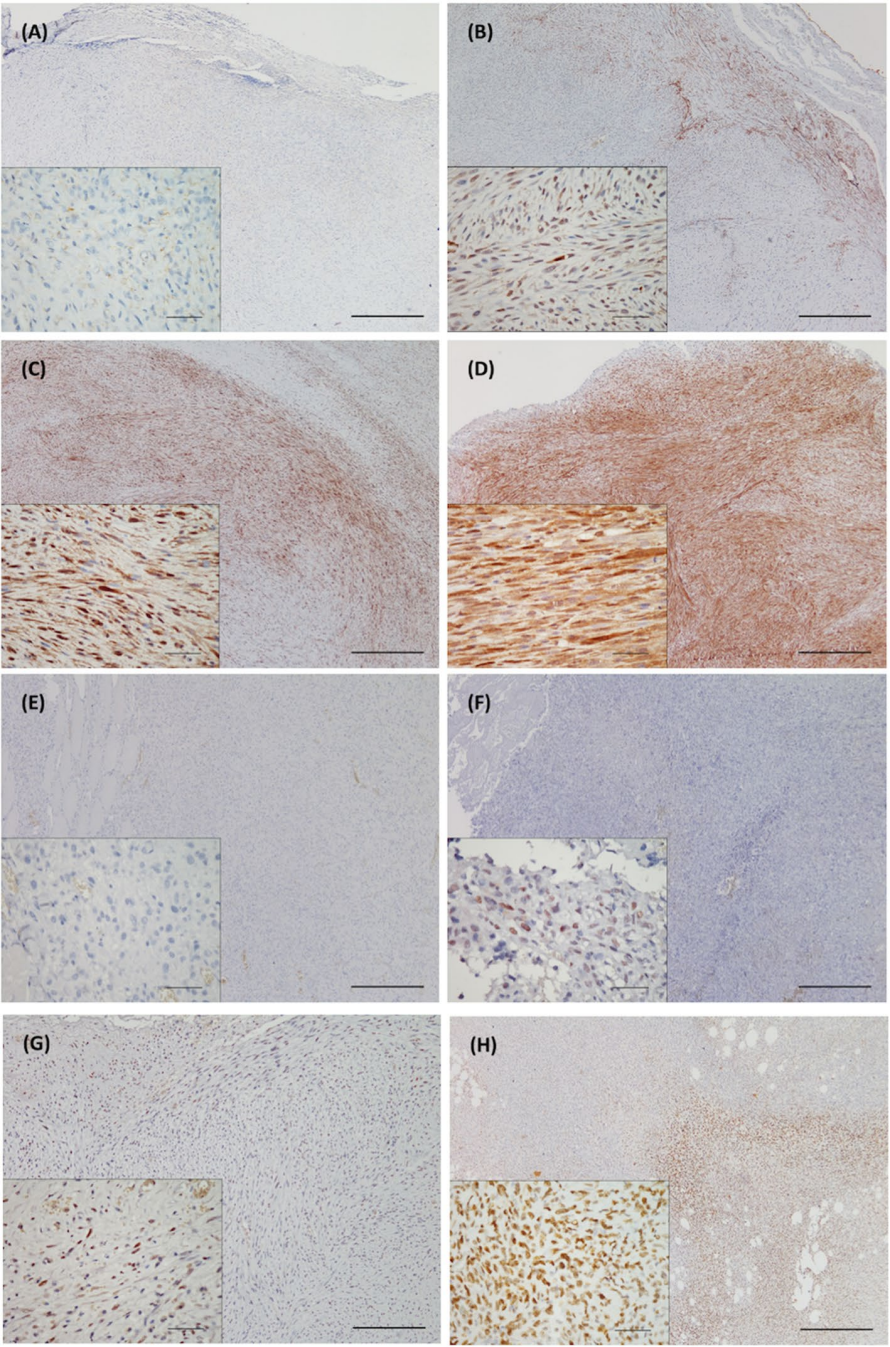
Detection of the expression of STAT3 (cytoplasmic) and phospho-STAT3 (Tyr705) by immunohistochemistry in formalin-fixed and paraffin-embedded (FFPE) tissue of feline injection site sarcomas. Images A-D represent the grade 0–3 expression of panSTAT3, respectively. Images E-H represent the grade 0–3 expression of phospho-STAT3 (Tyr705), respectively. The detailed grading criteria is as follows. Count 10 HPF for each section (at least 1000 cells), 0 = negative; 1 = less than 10% cells positive; 2 = 10–50% cells positive; 3 = more than 50% cells positive Scale bars = 500 μm; inset: scale bars = 50 μm

In this study, fourty-five FISS cases diagnosed at our institute during the period of 2014 and 2018 were included. According to the soft tissue sarcoma grading system [36], 11.2% (5/45), 44.4% (20/45), and 44.4% (20/45) of the FISSs were graded as I, II, and III, respectively (Table 1). While there was no detectable STAT3 and phosphor-STAT3 expression in the normal soft tissues surrounding FISSs, including skeletal and smooth muscles, adipose tissue, vessels, neuron, or connective tissues, our IHC staining revealed that approximately 88.9% (40/45) of the cases demonstrated cytoplasmic and/or nuclear positivity for STAT3 and 11.1% (5/45) of the cases were negative for STAT3 (score = 0) (Fig. 1). Cytoplasmic STAT3 expression with score 1, 2, and 3 were noted in 11.1% (5/45), 24.4% (11/45), and 53.3% (24/45) FISS cases, respectively; nuclear STAT3 expression with score 1, 2, and 3 were noted in 20% (9/45), 35.6% (16/45), and 26.7% (12/45) FISS cases, respectively. Approximately 82.2% (37/45) of the FISS cases showed cytoplasmic and nuclear double positivity for STAT3 (scores ranging from 1 to 3).

**Table 1:** Clinical data, tumor grading and immunohistochemical results of STAT3 and phospho-STAT3 (Try705) of 45 feline injection site sarcomas^a^

	Pathology number	Breed	Sex	Age (year)	Location	Grading	STAT3^b^ (cytoplasm/nucleus)	Phospho-STAT3^b^ (nucleus)
1	NTU2014-0003	Unknown	F	16	Dorsal neck	III	3/3	0
2	NTU2014-0015	Mixed	Fsp	11	Interscapula	III	3/2	1
3	NTU2014-0246	Mixed	Fsp	10	Back	II	3/3	2
4	NTU2014-0398	Mixed	Mc	9	Back	II	1/2	0
5	NTU2014-0427	Mixed	Fsp	11	Right thigh	III	2/3	1
6	NTU2014-0611	Mixed	Mc	6	Back	III	3/3	1
7	NTU2014-1505	Persian	Fsp	8	Interscapula	II	2/3	0
8	NTU2014-1771	Mixed	F	3	Interscapula	II	2/2	0
9	NTU2014-1978	Persian	Fsp	13	Back	III	3/1	0
10	NTU2014-2077	Mixed	M	8	Interscapula	II	3/3	0
11	NTU2014-2752	Mixed	Fsp	10	Back	I	1/1	1
12	NTU2014-2758	Mixed	Mc	10	Interscapula	II	2/2	0
13	NTU2014-2819	Mixed	Fsp	10	Back	III	3/2	1
14	NTU2014-2990	Mixed	M	3	Back	III	1/2	1
15	NTU2015-0387	Mixed	Mc	10	Back	II	2/1	0
16	NTU2015-0434	Mixed	Fsp	11	Left scapula	III	3/3	2
17	NTU2015-0913	Scottish Fold	Fsp	8	Dorsal neck	II	0/0	0
18	NTU2015-0942	Mixed	Mc	5	Back	I	3/2	0
19	NTU2015-1113	Mixed	F	12	Back	III	1/1	0
20	NTU2015-1101	Mixed	Mc	11	Back	II	1/1	1
21	NTU2015-1147	Mixed	Mc	11	Right scapula	II	0/0	0
22	NTU2015-1180	Mixed	Fsp	11	Dorsal neck	I	2/3	1
23	NTU2015-1522	Mixed	Mc	7	Right scapula	III	0/0	0
24	NTU2015-1838	Mixed	Fsp	8	Back	II	2/0	0
25	NTU2015-1873	Mixed	Mc	9	Interscapula	II	3/2	0
26	NTU2015-2125	Mixed	Fsp	9	Interscapula	II	3/2	0
27	NTU2015-2312	Mixed	Fsp	10	Dorsal neck	I	3/2	0
28	NTU2015-2410	Mixed	M	8	Back	II	0/0	0
29	NTU2015-2535	Persian	F	12	Back	III	3/2	1
30	NTU2015-3035	Mixed	Fsp	12	Interscapula	II	0/0	0
31	NTU2015-3397	Unknown	F	12	Dorsal neck	II	3/2	2
32	NTU2015-3449	Mixed	Mc	Unknown	Right scapula	II	2/1	1
33	NTU2016-2413	Mixed	Fsp	5	Back	I	3/2	2
34	NTU2018-1819	Mixed	Mc	10	Back	III	3/3	3
35	NTU2018-1944	Domestic short hair	Mc	7	Left trunk	III	3/3	3
36	NTU2018-2134	American short hair	Fsp	11	Right scapula	III	3/2	1
37	FISS-05	Mixed	Mc	6	Back	I	3/1	0
38	FISS-06	Persian	Mc	9	Back	II	3/2	1
39	FISS-07	Mixed	Mc	5	Right scapula	III	3/1	2
40	FISS-08	American short hair	Mc	10	Neck	III	3/1	1
41	FISS-10	Mixed	Fsp	10	Left scapula	II-III	2/0	0
42	FISS-12	Domestic short hair	Fsp	9	Left trunk	III	2/0	2
43	FISS-14 (NTU2018-1134)	Mixed	Mc	6	Left trunk	II	2/2	2
44	FISS-15	Domestic short hair	Mc	12	Right elbow	III	3/3	2
45	FISS-16	Mixed	Mc	7	Back	II	3/3	3

^a^ *M *Male; *Mc*, Male castrated, *F*, Female; *Fsp *Female spayed

^b^ Count 10 HPF for each section (at least 1000 cells), 0 = negative; 1 = less than 10% cells positive; 2 = 10-50% cells positive; 3 = more than 50% cells positive

The phospho-STAT3 (Tyr705) expression was variable in the FISS tissues (Fig. 1); approximately 53.3% (24/45) of the cases showed nuclear positivity for phospho-STAT3 (Tyr705), and 46.7% (21/45) of the cases were negative for phospho-STAT3 (Tyr705) (score = 0) under IHC staining. Nuclear phospho-STAT3 (Tyr705) expression with score 1, 2, and 3 were noted in 28.9% (13/45), 17.8% (8/45), and 6.7% (3/45) FISS cases, repsectively, According to Pearson’s correlation coefficient (r), the IHC scores of STAT3 and phospho-STAT3 (Tyr705) in FISSs were not significantly correlated with tumor grading, necrosis, and mitosis (*P* > 0.05).

### Correlation of the immunophenotypes of the primary FISS cells with their corresponding formalin-fixed and paraffin-embedded (FFPE) samples

**Fig. 2:**
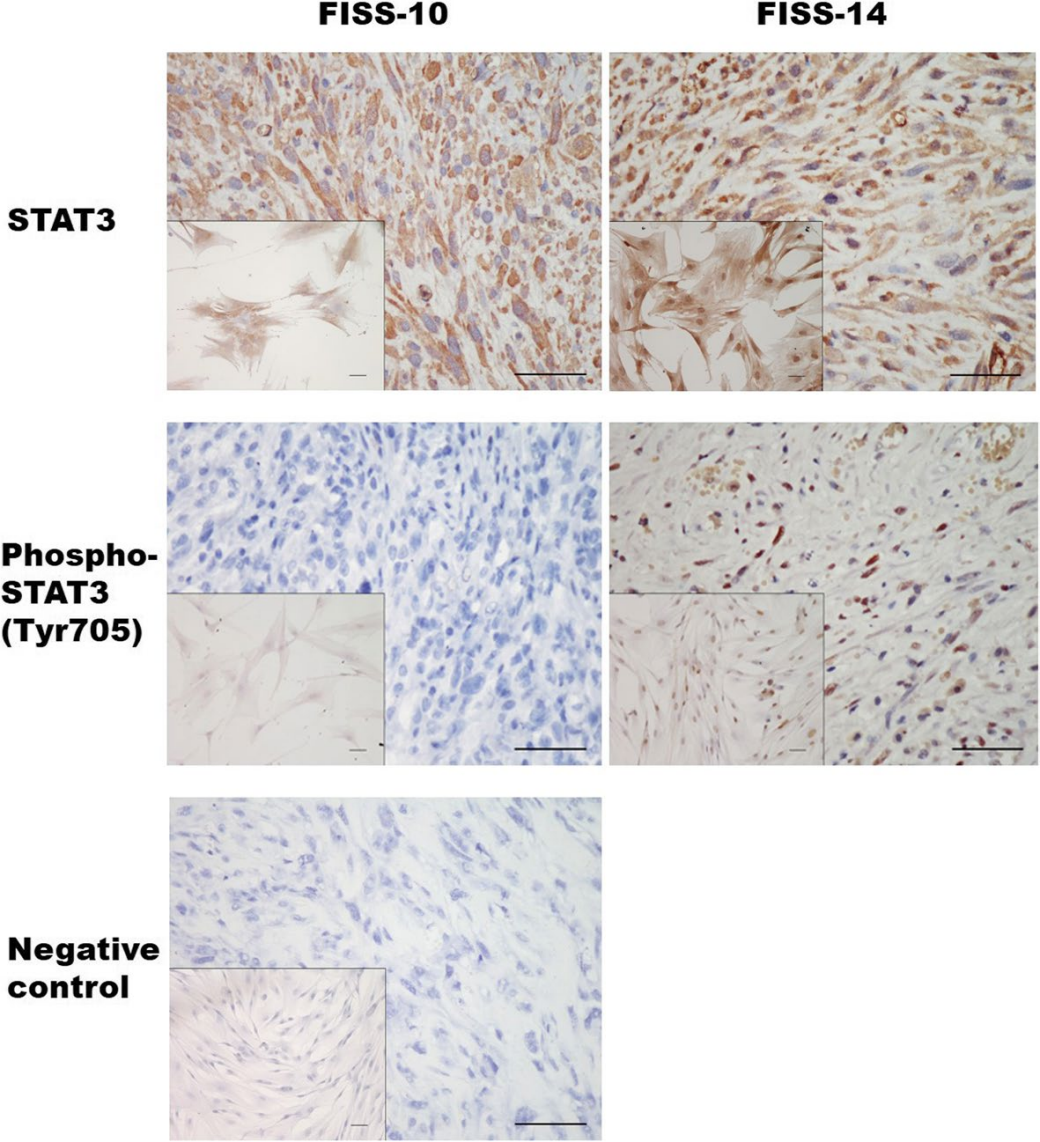
The correlation of immunophenotypes of feline injection site sarcoma (FISS) cells in formalin-fixed and paraffin-embedded (FFPE) tissue sections by immunohistochemical (IHC) and the corresponding primary cells derived from FISS by immunocytochemical (ICC) staining. Images show the expression of panSTAT3 and phospho-STAT3 (Tyr705) in FFPE tissues of FISS-10 and FISS-14 and the corresponding derived primary cells (inset). The substitution of the primary antibody with antibody diluent was performed in the negative control. Scale bars = 50 μm; inset: scale bars = 50 μm

In total, primary FISS cells were successfully obtained from the eight FISSs. To correlate the immunophenotypes of the primary FISS cells with their corresponding original FFPE samples, immunocytochemical (ICC) and IHC using a panel of nine different antibodies were performed in the eight primary FISS cells and the corresponding FFPE tissue sections, respectively. The results are shown in Fig. 2; Table 2. All primary FISS cells and their corresponding FFPE samples were positive for STAT3, NF-κB, and vimentin and negative for S100, Melan-A, and cytokeratin (CK). Based on these results, we confirmed that these primary FISS cells and FFPE samples were sarcomas of mesenchymal origin. Incompatible and variable staining results were observed for phospho-STAT3 (Tyr705), alpha-smooth muscle actin (α-SMA), and desmin. Most of the primary FISS cells and their corresponding FFPE samples were positive for α-SMA and negative for desmin, suggesting a myofibroblastic origin. Interestingly, most primary FISS cells were negative for phospho-STAT3 (Tyr705); however, their corresponding FFPE samples demonstrated immunoreactivity for phospho-STAT3 (Tyr705). Consistent ICC and IHC patterns were observed in the primary cells of FISS-10 and FISS-14 and their corresponding FFPEs. Both primary cells exhibited cytoplasmic STAT3 signals; however, different nuclear phospho-STAT3 (Tyr705) signals. These two primary cells were selected for in vitro LLL12 inhibition assays to compare the effects of LLL12 on FISS cells with or without nuclear phospho-STAT3 (Tyr705) signals. No detectable STAT3 and phosphor-STAT3 expression was noted in the cultured feline normal tissue cells (Supplementary Fig. 2).

**Table 2:** The results of IHC and ICC profiles of different FISS primary cells and their corresponding FFPE specimens

^a^IHC/^b^ICC profile								
	FISS-05	FISS-06	FISS-07	FISS-08	FISS-10	FISS-12	FISS-14	FISS-17
STAT3	+/+	+/+	+/+	+/+	+/+	+/+	+/+	+/+
Phospho-STAT3 (Tyr705)	**+/-**	**+/-**	**+/-**	**+/-**	-/-	+/+	+/+	**+/-**
^d^NF-κB p65	+/+	+/+	+/+	+/+	+/+	+/+	+/+	+/+
^c^α-SMA	+/+	+/+	**+/-**	+/+	+/+	**-/+**	+/+	+/+
S100	-/-	-/-	-/-	-/-	-/-	-/-	-/-	-/-
Melan-A	-/-	-/-	-/-	-/-	-/-	-/-	-/-	-/-
^e^CK	-/-	-/-	-/-	-/-	-/-	-/-	-/-	-/-
Vimentin Desmin	+/+ -/-	+/+ -/-	+/+ -**/+**	+/+ -/-	+/+ -/-	+/+ -/-	+/+ -/-	+/+ -/-

^a^*IHC* immunohistochemistry, ^b^*ICC* immunocytochemistry, *FFPE* formalin-fixed and paraffin-embedded

^c^*α-SMA* alpha-smooth muscle actin

^d^*NF-κB* nuclear factor-kappa B

^e^*CK* cytokeratin

- = negative; + = more than 5% cells positive

### STAT3 inhibitor (LLL12) inhibits the proliferation of FISS cells with a dose-dependent effect

**Fig. 3:**
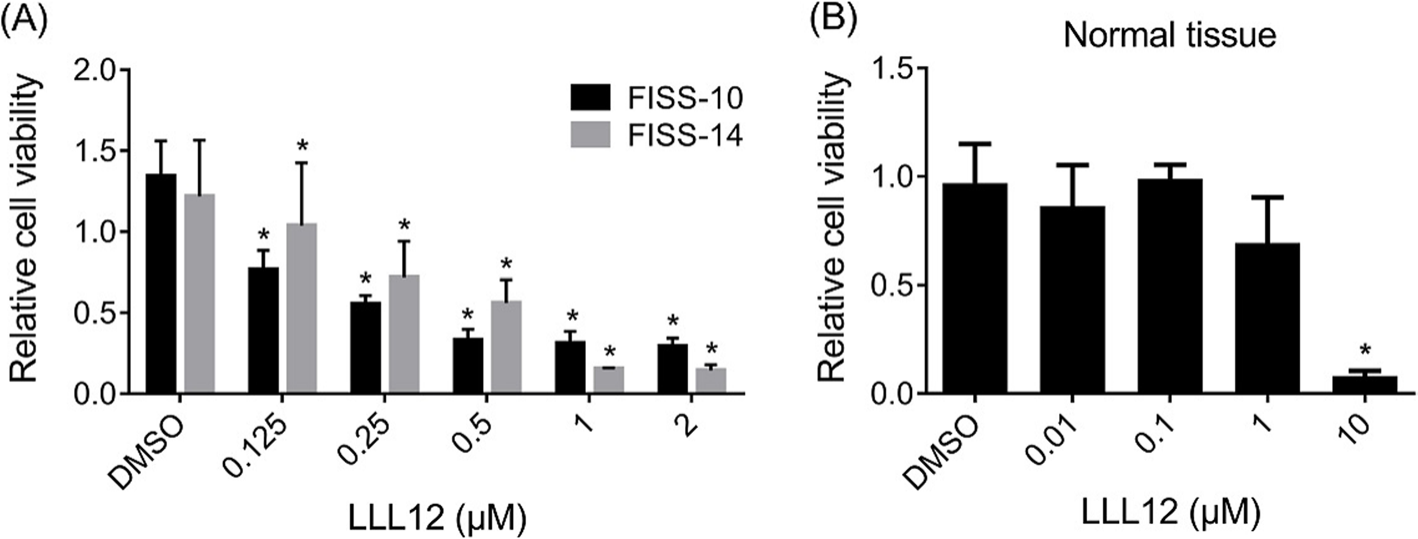
The effect of 5-hydroxy-9,10-dioxo-9,10-dihydroanthracene-1-sulfonamide (LLL12) on the cell viability of the primary cells derived from feline injection site sarcomas (FISSs) and feline normal soft tissues evaluated at 72 h of treatment. The FISS-10 and FISS-14 primary cells (**A**) and the normal feline soft tissue cells (**B**) were treated with different concentrations of LLL12 in triplicate. The inhibitory effects of LLL12 on cell proliferation were evaluated at 24, 48 and 72 h of LLL12 treatment by the Alamar Blue assay. DMSO was used for dissolving the LLL12 and was used as a vehicle control. Data are present as mean ± SD. *Statistically significant from the controls (*P* < 0.05)

To assess the role of STAT3 in tumorigenesis, FISS-10 and FISS-14 primary cells exhibiting consistent ICC and IHC patterns with their corresponding FISS cells in FFPE were treated with the STAT3 inhibitor LLL12. Cell viability was evaluated using the AlamarBlue assay. Interestingly, LLL12 was able to inhibit the proliferation of both FISS-10 and FISS-14 primary cells in a dose-dependent manner (Table 3; Fig. 3A). Compared with the cells in the control group, significant growth inhibition was observed at a concentration of 0.125 µM in FISS-10 and FISS-14 cells with no obvious time differences at 24, 48, and 72 h of treatment. Normal feline soft tissue cells were treated with the same formula, and significant growth inhibition was observed only at a concentration of 10 µM in the absence of a dose-dependent inhibitory effect (Fig. 3B). The IC_50_ concentrations of FISS cells and normal feline soft tissue cells are shown in Table 4. Compared to the normal feline soft tissue cells with IC_50_ values of 1.697 µM at 24 h of treatment and 1.665 µM at 72 h of treatment, both FISS-10 and FISS-14 cells exhibited greater vulnerability to the treatment. At 24 h of treatment (Table 4), the FISS-10 and FISS-14 cells had 5.6 and 3.4 fold lower IC_50_ values than those of normal soft tissue cells, respectively. The differences increased slightly to 7.7 fold and 3.7 fold lower IC_50_ values in FISS-10 and FISS-14 than those of normal soft tissue cells after 72 h of treatment, respectively. Accordingly, the FISS-10 cells were more sensitive to LLL12 treatment than the FISS-14 cells.

**Table 3:** The result of cell survival rate (% reduction of AlamarBlue) of different primary cells derived from feline injection site sarcomas (FISSs) following treatment with different concentrations of 5-hydroxy-9,10-dioxo-9,10-dihydroanthracene-1-sulfonamide (LLL12) for 1-3 days

	FISS-10						FISS-14					
LLL12	day 1		day 2		day 3		day 1		day 2		day 3	
(μM)	Mean	SD	Mean	SD	Mean	SD	Mean	SD	Mean	SD	Mean	SD
2	31.892	0.186	23.754	0.242	32.665	0.177	11.279	0.585	17.964	0.399	14.257	0.482
1	37.898	0.594	23.494	0.151	32.586	0.160	15.272	0.580	16.165	0.297	14.692	0.244
0.5	39.305	1.025	25.812	0.562	34.366	0.192	41.122	2.812	69.744	3.587	57.243	3.394
0.25	60.345	1.347	49.953	0.939	56.049	0.736	46.300	14.753	79.969	16.165	88.842	3.645
0.125	71.976	1.316	67.882	2.125	90.040	1.662	62.514	10.629	139.298	1.913	109.572	3.076
DMSO	122.028	1.679	121.097	3.457	159.510	4.100	104.645	6.453	161.766	3.668	98.890	22.530
0	100.000	1.952	100.000	3.845	100.000	9.086	100.000	2.012	100.000	21.695	100.000	9.935

**Table 4:** The IC_50_ of 5-hydroxy-9,10-dioxo-9,10-dihydroanthracene-1-sulfonamide (LLL12) for primary cells of feline injection site sarcoma (FISS) and feline normal soft tissue (FNST) following 24 or 72 hours of incubation

	IC_50_ of LLL12 (μM)	
Cell	24 hours	72 hours
FISS-10	0.2999	0.2231
FISS-14	0.5061	0.4510
FNST	1.697	1.665

### Inhibition of the migration of FISS primary cells by LLL12 using the wound healing assay

**Fig. 4:**
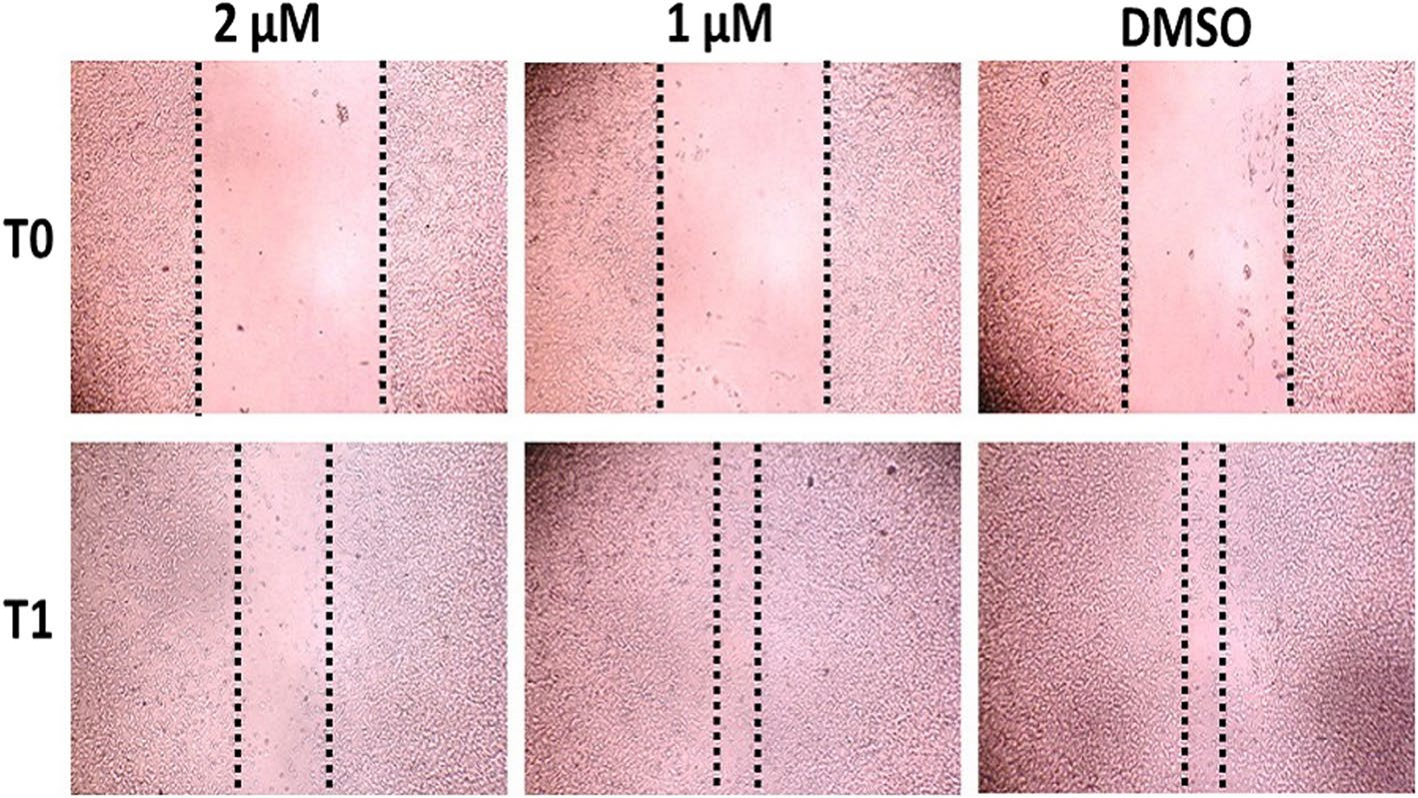
The result of wound healing assay of primary cells derived from feline injection site sarcoma (FISS) treated with 5-hydroxy-9,10-dioxo-9,10-dihydroanthracene-1-sulfonamide (LLL12) at different concentrations. The primary cells of FISS-10 were treated with different concentrations of LLL12. The width of the wounds was measured and photographed before the LLL12 treatment (T0) and at 24 h of treatment (T1). DMSO was used as the vehicle control

Since the FISS-10 primary cells exhibited the highest sensitivity to LLL12 treatment and a limited number of FISS-14 primary cells were collected, wound healing was performed in FISS-10 cells. A dose-dependent reduction in cell migration was identified under 0.25-2 µM of LLL12 treatment. Under 2, 1, and 0.5 µM LLL12 treatment, the migration distances of FISS-10 cells were reduced to 42.1–80% of the migration distance of DMSO-treated cells. Significant inhibition by LLL12 was detected at a concentration of 2 µM in FISS-10 primary cells (*P* < 0.05) (Fig. 4; Table 5). There was no significant inhibition at concentrations of 0.125, 0.25, 0.5, and 1 µM.

**Table 5:** The result of migration inhibition of 5-hydroxy-9,10-dioxo-9,10-dihydroanthracene-1-sulfonamide (LLL12) on the primary cells derived from feline injection site sarcoma (FISS) following incubation with different concentrations for 24 hours evaluated by cell migration assay^a^

LLL12 (μM)	FISS-10		
	Average	% of control	SD
2	409.831	42.1*	2.611
1	721.171	80.9	75.594
0.5	682.277	80.5	31.343
0.25	849.18	97.2	83.353
0.125	753.586	81.4	19.806
0	871.470	97.2	40.710
DMSO	942.025	100	5.815

*Statistically significant from the DMSO control (*P* < 0.05)

^a^ The migration of FISS cells was determined by the distance (μm) of cells that cross into the wound area from their reference point at time zero. The measurement included at least 50 readings of distance for each sample and each experiment was done in triplicate

## Discussion

In the present study, the roles of STAT3 and phospho-STAT3 (Tyr705) in the tumorigenesis of FISSs were investigated. We have demonstrated that concurrent cytoplasmic and/or nuclear signals of STAT3 and nuclear signals of phospho-STAT3 (Tyr705) were detected in 82.2% and 53.3% of FISS cases, respectively. While the IHC scores of both STAT3 and phospho-STAT3 (Tyr705) in FISSs were not significantly correlated with tumor grading (*P* > 0.05), the high expression of both STAT3 and phospho-STAT3 (Tyr705) in FISSs suggests that disruption of the Janus kinase (JAK)-STAT3 signaling pathway may be a potential therapy for FISSs. Using two primary cells derived from FISSs (FISS-10 and FISS-14) showing consistent ICC and IHC patterns as their parental FFPE tissues, a dose-dependent inhibitory effect on the growth and migration ability of FISS cells after treatment with the STAT3 inhibitor LLL12 was confirmed. These results suggest that JAK-STAT3 signaling pathway may play an important role in the sarcomagenesis of FISS.

The expression of pan-STAT3 in FISSs has been demonstrated using pan-STAT3 antibody [31]. In a previous study, cytoplasmic STAT3 expression was detected in 83.33% (20/24) of cats treated with doxorubicin before surgery, and in 100% (15/15) of cats that received surgical treatment alone. Doxorubicin treatment has been suggested to significantly decrease STAT3 expression as compared with cases treated by surgery alone [31]. In the present study, we demonstrated a high cytoplasmic expression level of STAT3 in 88.9% of FISSs, which is similar to the previous research. Unfortunately, due to the current research is mainly based on a retrospective investigation and several limitations owing to the patient information was originally not designed to collect data for research, some information is bound to be missing. We are unable to track back the history of chemotherapy of all animals used in this study for better correlating the role of chemotherapy on STAT3 expression in FISS. In addition to the potential effect of doxorubicin before surgery on the expression of STAT3 in FISS, factors such as individual variations of investigated animals in different studies and different procedures and conditions during FFPE preparation, such as post-mortem delay, fixation time, paraffin, storage time in paraffin, storage temperature, and age of the cut sections have been reported to influence the outcome of IHC [37] since most of FFPE tissue sections used in the present study were derived from tissue blocks collected during 2014 to 2018. Furthermore, regarding the pan-STAT3 antibody used for STAT3 detection could not differentiate STAT3 from phospho-STAT3 (Tyr705), in general, the majority of STAT3 located in the cytoplasm is in an inactive form [38, 39], and inactive STAT3 could also constitutively shuttle between the cytoplasm and nucleus [40]. Detection of phosphorylated STAT3 using western blotting confirmed antibody was performed in our study to specifically detect the activated form of STAT3. To our knowledge, this is the first report on the detection of phospho-STAT3 (Tyr705) in FISS. We demonstrated that 53.5% (24/45) of the cases expressed nuclear phospho-STAT3 (Tyr705) signals. There were more immunolabelled neoplastic cells at the periphery of the neoplasms than in the central region. This phenomenon may result from tissue necrosis in the central region. It is generally agreed that the formation of FISS is highly associated with chronic inflammation and wound healing. Therefore, the high expression of cytoplasmic STAT3 in FISS is reasonable because this transcription factor is commonly activated by cytokines of the IL-6 family, which are produced by macrophages and stromal fibroblasts during chronic inflammation [23].

Since STAT3 is involved in many different signaling pathways, interpretation of STAT3 activation is a complicated event. Herein, we focused on the JAK-STAT signaling pathway and, more specifically, the expression of phospho-STAT3 (Tyr705). Our results demonstrated that phospho-STAT3 was expressed in more than half of FISS cases. Phospho-STAT3 (Tyr705) expression is closely correlated with the histological grading and intratumor microvessel density in HCC [33].Additionally, STAT3 activation is correlated with a better or worse prognosis depending on the tumor type [41]. The involvement of phospho-STAT3 (Tyr705) in cell proliferation, metastasis, and angiogenesis, and its correlation with prognosis in FISS cases warrant further investigation.

In our study, the expression of STAT3 and/or phospho-STAT3 (Tyr705) in FISSs did not correlate with tumor grading. These results differ from those of feline mammary gland tumors [27, 32]. Unlike FISSs, grading systems for feline mammary gland tumors [42] have been well studied. However, there are no widely accepted criteria for histological grading of FISS. We speculated that the failure to correlate the expression of STAT3 with the pathological grading in FISSs may be due to the soft tissue sarcoma grading system used in this and previous studies [20, 43–45] was inappropriate for FISS since most of the cases were graded as 2–3 due to the high score allocated for necrosis. The correlation between grading and recurrence was also not significant [44]. An appropriate grading system for FISS should be further modified by placing less emphasis on the necrosis score.

In this study, eight FISS-derived primary cells were established and demonstrated consistent positive immunoreactivity for STAT3, NF-κB, and vimentin; however, negative for S100, Melan-A, and CK with their corresponding FFPE samples. The cytological appearance of the FISS primary cells combined with diffuse strong vimentin reactivity supports a mesenchymal origin. The lack of immunoreactivity for S100, Melan-A, and CK excluded the neural crest, myoepithelial, melanocytic, and epithelial origins [46]. However, incompatible and variable results were observed for phospho-STAT3 (Tyr705), α-SMA, and desmin IHC/ICC staining. Many tumor cells that display constitutive STAT3 activation in vivo rapidly lose STAT3 phosphorylation once being placed in a culture medium without neighboring immune or stromal cells [47]. As the formation of phospho-STAT3 (Tyr705) requires chronic cytokine stimulation from neighboring stromal or inflammatory cells [38], the different in vitro and in vivo expression patterns of phospho-STAT3 (Tyr705) may result from different tumor microenvironments.

In the present study, all eight FISS-derived primary cells and their parental FFPE samples showed immunoreactivity for STAT3 and NF-κB. Nuclear expression of NF-κB was detected in 83.3% of FISS cases in our previous study [20]. NF-κB and STAT3 control both distinct and overlapping groups of anti-apoptotic, pro-proliferative, and immune response genes during tumorigenesis. They cooperate to promote the development and progression of epithelial tumorigeneses, such as colon, gastric, and liver cancers [48]. Their high expression in FISS suggests that they may play an important role in chronic inflammation and tumor progression; however, the effects on the tumor, immune, and inflammatory cells, and the interactions of these two transcriptional factors require further investigation.

**Fig. 5:**
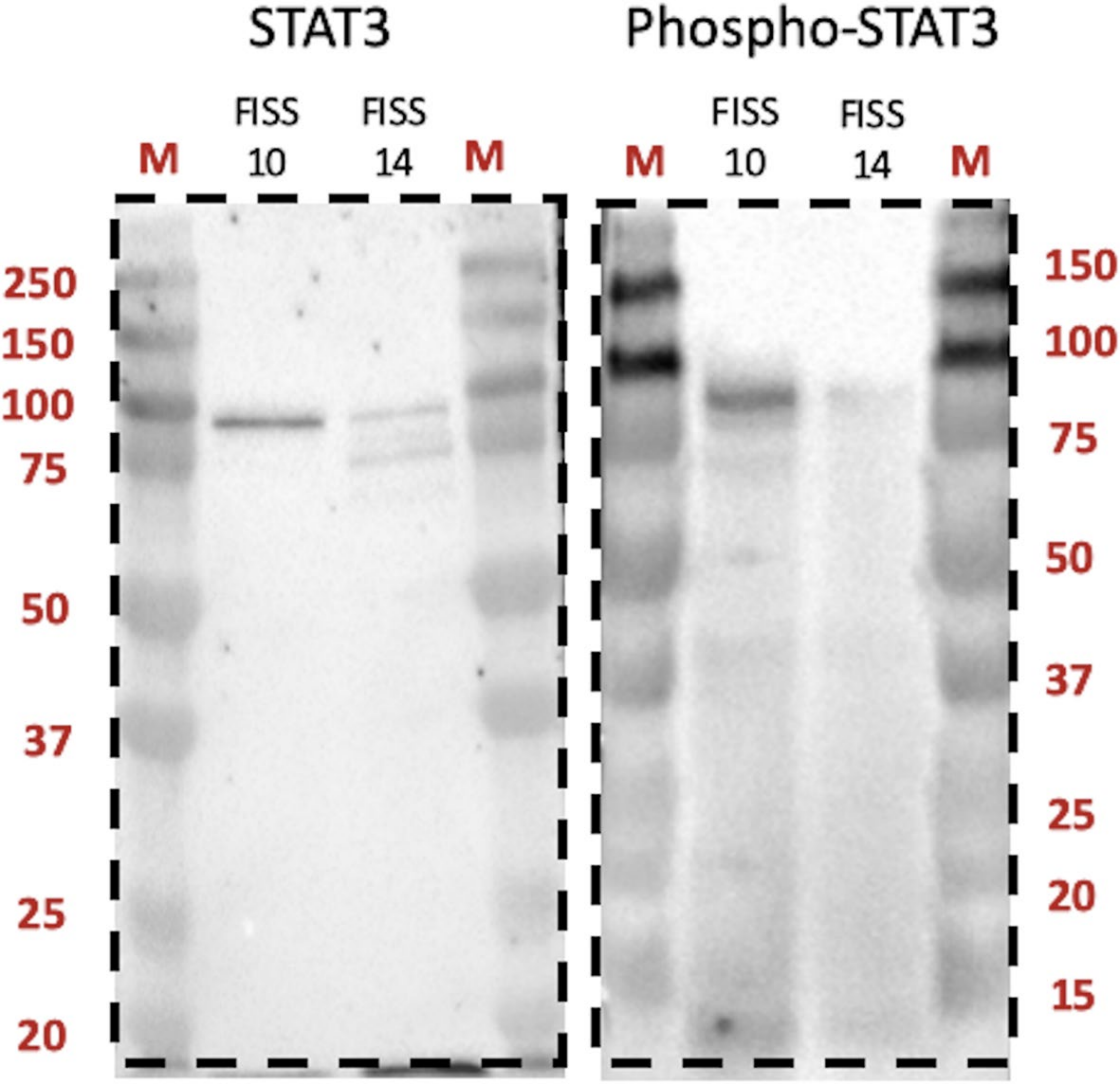
Western blot for the detection of STAT3 and phospho-STAT3 (Tyr705) using mouse monoclonal STAT3 (124H6, Cell Signaling Technology) and rabbit monoclonal phospho-STAT3 (Tyr705) (D3A7, Cell Signaling Technology) antibody in the cell lysates of primary cells of two feline injection site sarcomas (FISSs). Distinct bands of size about 85 kDa (marked with arrowhead) were detected. M: markers. The blots in this figure were cropped before hybridization and the whole blots were presented in Supplementary Fig. 1

The two FISS-derived cells (FISS-10 and FISS-14) exhibiting consistent ICC and IHC patterns with their corresponding FFPE specimens were treated using the STAT3 inhibitor LLL12. A dose-dependent inhibitory effect was noted in both the FISS-derived primary cells treated with LLL12, while the inhibitory effect was absent in LLL12-treated feline normal soft tissue cells. LLL12 is a potent and selective inhibitor of STAT3 phosphorylation. A computer model with docking simulation showed that LLL12 binds directly to the phosphoryl tyrosine 705 binding site of the STAT3 monomer [49]. These feline normal soft tissue cells were negative for STAT3, which may elucidate why they are not vulnerable to treatment. Similarly, normal canine osteoblasts are also comparatively resistant to the anti-proliferative effects of LLL12 [50]. LLL12 significantly reduces cell proliferation in three feline OSCC cell lines, and the most sensitive one correlates with the highest level of basal STAT3 phosphorylation [29]. However, in the present study, we failed to demonstrate a correlation between the expression level of phosphorylated STAT3 and cell sensitivity to the STAT3 phosphorylation inhibitor LLL12. We have demonstrated that the primary cells derived from both FISS − 10 and FISS-14 were sensitive to LLL12 treatment regardless of STAT3 expression detected by IHC or ICC. Interestingly, we observed that the FISS-10-derived primary cells, which did not show immunoreactivity of phospho-STAT3 (Tyr705) in both IHC and ICC staining, were even more sensitive to LLL12 treatment than the FISS-14 primary cells. These results indicate that both FISS-derived primary cells are highly dependent on STAT3 signaling. To explain the result, we have performed western blotting, the more sensitive method than ICC and IHC, and demonstrated that STAT3 and phospho-STAT3 (Tyr705) were detected in comparable level in both FISS-10 and FISS-14 primary cells (Fig. 5). Since STAT3 is generally constitutively expressed, the absence of nuclear or phosphorylated STAT3 expression in FISS-10 might be due to the insensitivity of IHC and ICC. This may also be due to the natural dephosphorylation of nuclear phospho-STAT3 (Tyr705) [51] in FISSs or inappropriate specimen handling and slow fixation [52]. Our results suggest that western blot should always be included for characterize the expression level of STAT3 and phospho-STAT3 (Tyr705) in tumor cell studies.

## Conclusions

Feline injection site sarcomas are aggressive neoplasms with the incidence about 1/1,000–10,000 in vaccinated cats. The high expression rates of STAT3 and phospho-STAT3 (Tyr705) in FISSs and the in vitro reduction in proliferation and tumor migration in FISS primary cells by the STAT3 phosphorylation (Tyr705) inhibitor in the present study suggest that the STAT3 may play an important role in the tumorigenesis of FISS and could be a potential therapeutic target for FISSs.

## Methods

### Case collection

A total of 45 clinical feline cases with a histological diagnosis of FISS referred to the Graduate Institute of Molecular and Comparative Pathobiology, National Taiwan University (NTU) from 2014 to 2018 were used in the present study. Data tabulated from the database were pathology number, breed, sex, age, tumor location, and sarcoma grading (Table 1). Histopathology slides and formalin-fixed and paraffin-embedded (FFPE) tissue sections were retrieved for this study.

### Immunohistochemical staining for FFPE tissues

Four-micrometer-thick FFPE slides were deparaffinized using xylene, rehydrated with decreasing concentrations of ethanol, and rinsed with Tris-buffered saline, 0.1% Tween 20 (TBST). Antigen retrieval was performed by boiling the slides in an EDTA-based retrieval buffer (Trilogy™; Cell Marque, Rocklin, CA, USA) in a microwave (EZ-Retriever® System; BioGenex Laboratories Inc., San Ramon, CA, USA) at 95 °C for 10 min. The slides were then incubated with 10% normal goat serum (Dako, CA, USA) diluted in phosphate-buffered saline (pH 7.4) at room temperature (RT) for 30 min to block non-specific binding sites. Endogenous peroxidase activity was removed by incubating the slides with 3% and 0.5% hydrogen peroxide (KYB, New Taipei City, Taiwan) for 15 min for STAT3 and phospho-STAT3 (Tyr705) detection, respectively. Expressions of STAT3 and pSTAT3 were detected by the primary antibody to STAT3 (clone 124H6; Cell Signaling Technology, Danvers, MA, USA) at 1 in 400 dilutions in 10% normal goat serum for 1 h at room temperature and the rabbit monoclonal antibody to phospho-STAT3 (Tyr705) (clone D3A7; Cell Signaling Technology) at 1 in 200 dilutions in 10% normal goat serum overnight at 4 °C. Dilutions of the other antibodies used in this study are listed in Tables 6 and 7. For detection, the sections were treated with Dako Real Envision-HRP (rabbit/mouse) (Dako Denmark A/S, Glostrup, Denmark) for 40 min at room temperature. After washing in TBST, the reaction products were visualized with 2% diaminobenzidine (Real DAB + chromogen, Dako Denmark A/S) for 3 min at room temperature. The slides were finally counterstained with Mayor’s hematoxylin solution (Muto Pure Chemicals, Tokyo, Japan) for 40 s. The primary antibody was substituted with the antibody diluent as a negative control.

**Table 6:** Primary antibodies, sources and dilutions

Antibody	Manufacturer code	Type/species	Dilution
STAT3	Cell Signaling 9139	Monoclonal/mouse	1:400
Phospho-STAT3 (Tyr705)	Cell Signaling 9145	Monoclonal/rabbit	1:200
NFκB p65	Abcam ab86299	Polyclonal/rabbit	1:200
α-SMA	Dako M0851	Monoclonal/mouse	1:200
S100	Leica S100P-L-CE	Polyclonal/rabbit	1:100
Melan-A	Leica MELANA-L-CE	Monoclonal/mouse	1:50
Cytokeratin	Genemed 61-0022	Monoclonal/mouse	1:100
Vimentin	Leica VIM-V9-L-CE	Monoclonal/mouse	1:100
Desmin	Dako M0760	Monoclonal/mouse	1:200

**Table 7:** Clinical data and pathological features of the 2 feline injection site sarcomas (FISSs) used for the establishment of primary cells

	FISS-10	FISS-14 (NTU2018-1134)
Breed	Mixed	Mixed
Sex/Age(years)	Spayed female/10y	Castrated male/6y
Tumor size	<5cm	>5cm
Location	Left scapula	Left trunk
Pathological diagnosis	Sarcoma	Fibrosarcoma
Grading	II-III	II
^cb^IHC/^da^ICC profile		
^e^STAT3	2/+	2/+
^f^Phospho-STAT3 (Tyr705)	0/-	2/+

^a^ICC criteria: - = negative; + = more than 5% cells positive

^b^IHC criteria: count 10 HPF for each section (at least 1000 cells), 0 = negative; 1 = less than 10% cells positive; 2 = 10-50% cells positive; 3 = more than 50% cells positive

^c^*IHC* immunohistochemistry

^d^*ICC* immunocytochemistry

^e^*STAT3* signal transducer and activator of transcription 3

^f^Phospho-STAT3 (Try705) = signal transducer and activator of transcription 3 phosphorylated at tyrosine 705

### Histopathological and immunohistochemistry scoring criteria

A soft-tissue sarcoma grading system [36] was used for tumor grading in this study. The sarcomas were scored from 1 to 3 for cell differentiation (1 = well-differentiated; 2 = poorly differentiated but with defined histotype; 3 = undifferentiated); mitotic rates (1 = less than nine mitoses per 10 high power fields; 2 = 10–19 mitoses per 10 high power fields; 3 = more than 20 mitoses per 10 high power fields), and necrosis (1 = no necrosis; 2 = necrosis in less than 50% of the total area; 3 = necrosis in more than 50% of the total area). Total scores ≤ 3 were designated as grade I; 4–5 were designated as grade II, and scores ≥ 6 were designated as grade III.

For IHC scoring, a semiquantitative method reported by Petterino et al. (2009) was modified and applied in this study. The tissue sections were evaluated by light microscopy to determine the anti-STAT3 and anti-phospho-STAT3 (Tyr705) positivity based on the cellular location of positive immunolabelling, cytoplasmic or nuclear for STAT3, and nuclear for phospho-STAT3 (Tyr705). Positive cells were counted in 10 high power fields (HPF) for each tissue section. At least 1000 cells were analyzed. Briefly, 0, absence of immunoreaction; 1, less than 10% positive cells; 2, 10–50% positive cells; and 3, more than 50% positive cells.

### Primary FISS cells isolation and cultivation

To obtain the primary neoplastic FISS cell lines, wedge biopsies of masses developed at the injection site with a history of vaccination of cats were collected immediately after the surgery. For each case, a proportion of the mass was submitted for histopathological examination, and the remaining proportion was used for primary cell establishment. These cases were histologically confirmed as FISS. Explant culture was performed to establish primary FISS cells according to our previous study [20]. The tumor tissue was washed with phosphate-buffered saline (PBS) (Gibco, Grand Island, NY, USA)–3–4 times until the tissue became white in appearance, cut into numerous 1-mm^3^ fragments, and placed into a 75T sterile tissue culture flask (Corning, Corning, NY, USA) containing 15 mL of Dulbecco’s modified Eagle’s medium (DMEM, Gibco) supplemented with 20% fetal bovine serum (FBS, Gibco) 250 ng/mL Amphotericin B, 100 U/mL Penicillin, and 100 µg/mL Streptomycin (Gibco). Regarding the primary normal feline soft tissue, small pieces of soft tissues including fibrous connective tissue and skeletal muscle remote from the tumor site were aseptically removed and cut into small fragments. The tissues were washed with PBS (Gibco)–3–4 times and then digested with 0.05% trypsin (Gibco) for 10 min. The suspended pellet mixture was filtered through a pre-wet 100 μm strainer (CORNING) and the supernatant was collected, centrifuged, and re-suspended in DMEM (Gibco) containing 20% FBS (Gibco) and 250 ng/mL Amphotericin B, 100 U/mL Penicillin, and 100 µg/mL Streptomycin (Gibco). This suspension was placed in a 75T sterile tissue culture flask (Corning). The culture flasks of normal feline soft tissue and the tumor cells were placed in a 37ºC, humidified, 5% CO_2_ cell culture incubator for approximately 14 days, and the media were refreshed every 3 days. All functional tests were completed within a maximum of the first three passages of the primary cells.

### Validation of antibodies

Cross-species antibodies (Table 6) including mouse anti-human STAT3 (clone 124H6; Cell Signaling Technology, Danvers, MA, USA), rabbit anti-mouse phospho-STAT3 (Tyr705) (clone D3A7; Cell Signaling Technology, Danvers, MA, USA), rabbit anti-human NF-κB p65 (clone ab86299; Abcam, Cambridge, MA, USA), mouse anti-human α-SMA (clone 1A4; Dako, Glostrup, Denmark), mouse anti-human desmin (clone D33; Dako, Glostrup, Denmark), mouse anti-human cytokeratin (CK) (clone AE1/AE3; Genemed Biotechnologies, Torrance, CA, USA), mouse anti-human vimentin (clone V9; Leica Biosystems, Buffalo Grov, IL, USA), mouse anti-human Melan-A (clone A103; Leica Biosystems, Buffalo Grov, IL, USA), and rabbit anti-human S100 (polyclonal; Leica Biosystems, Buffalo Grov, IL, USA) were used in the present study for characterizing the phenotypes of the FFPE sections and their corresponding primary FISS cell lines. Antibodies for detecting α-SMA, desmin, CK, vimentin, Melan A, and S100 are commonly used for pathological diagnosis in veterinary medicine. The anti-NF-κB p65 antibody, clone ab86299 (Abcam), was characterized and used in our previous study on FISS [20]. Mouse anti-human STAT3 (clone 124H6; Cell Signaling Technology) and rabbit anti-mouse phospho-STAT3 (Tyr705) (clone D3A7; Cell Signaling Technology) have previously been used in feline mammary gland tumor studies [32], and the specificity of both antibodies was confirmed by western blotting in FISS-10 and FISS-14 primary cells (Fig. 5 and Supplementary Fig. 1).

### Preparation of primary FISS cell lysates

To determine the expression of STAT3 and phospho-STAT3 (Tyr705) in FISS-10 and FISS-14 primary cells, western blotting was performed. The primary cells in a 75T tissue culture flask (Corning) were washed twice with PBS (Gibco), trypsinized with 1 mL of StemPro Accutase Cell Dissociation Reagent (Accutase™, Gibco), and centrifuged at 1000 rpm for 8 min. The supernatant was then discarded. Protein extraction was achieved by incubating the cells in 1 mL of RIPA lysis buffer (VWR Chemicals, Sohn, Ohio, USA) on ice, mixed with cOmplete™ EDTA-free protease inhibitor cocktail (Roche Molecular Biochemicals, Laval, Quebec, Canada), and PhosSTOP™ phosphatase inhibitor (Roche Molecular Biochemicals) for 15 min followed by sonication of the cells in iced water for 20 min. The solution was then centrifuged at 13,000 rpm for 10 min, and the supernatant was stored in aliquots at -20 °C until use.

### Western blotting

Equal amounts of protein were mixed with an appropriate volume of lithium dodecyl sulfate loading buffer (Invitrogen) in the presence of a reducing agent (50 mM DTT) (Pierce Biotechnology), and the samples were heated in a dry bath at 65 °C for 10 min. The proteins were then separated on a 10% sodium dodecyl sulfate-polyacrylamide gel (10% resolving gel and 5% stacking gel), blotted onto a polyvinylidene difluoride membrane (Bio-Rad, Hercules, CA, USA), and blocked with BlockPRO™ 1 Min Protein-Free Blocking Buffer (Energenesis Biomedical, Taipei City, Taiwan) for 3 min. The blots were probed with antibodies against STAT3 (1:1000 dilution in blocking buffer; Cell Signaling Technology) and phospho-STAT3 (Tyr705) (1:2000 dilution in blocking buffer; Cell Signaling Technology) for 1 h at room temperature and then washed three times with PBS (Gibco). The blots were subsequently incubated using horseradish peroxidase-conjugated secondary anti-rabbit antibody (1:10000 dilution in blocking buffer; Jackson ImmunoResearch Laboratories, West Grove, PA, USA). The protein bands were visualized using the Clarity™ Western ECL Blotting Substrate (Bio-Rad) and detected using the ChemiDoc™ Imaging System (Bio-Rad).

### Immunocytochemistry staining for FISS cells

ICC staining was used to correlate the phenotypes of primary cell cultures with those of their corresponding FFPE tissue sections. The neoplastic cells and primary cells derived from feline normal soft tissue were fixed in 1:1 100% acetone/100% methanol fixative (Merck, Darmstadt, Germany) for 20 min at -20 °C. The fixative was aspirated, and the cells were rinsed twice with PBS. Except for the antibody used for phospho-STAT3 (Tyr705) detection, all antibodies were diluted to their working concentration as listed in Table 6 and applied to the cells for 1 h at room temperature. The antibody specifically targeting phospho-STAT3 (Tyr705) was first diluted to 1:200 and applied to the cells overnight at 4 °C. After the binding of primary antibodies, the cells were washed with PBS 6–10 times and treated with Dako Real Envision-HRP (rabbit/mouse) (Dako Denmark A/S, Glostrup, Denmark) for 60 min at room temperature. After washing with PBS 6–10 times, the reaction products were visualized with 2% diaminobenzidine (Real DAB + chromogen, Dako Denmark A/S, Glostrup, Denmark) for 3 min at room temperature. The cells were counterstained with hematoxylin (Muto Pure Chemicals, Tokyo, Japan) for 10 s. Negative controls were created by omitting the primary antibodies.

### Reagents

The STAT3 phosphorylation (Tyr705) inhibitor, 5-hydroxy-9,10-dioxo-9,10-dihydroanthracene-1-sulfonamide (LLL12; BioVision Milpitas, CA, USA), a non-peptide, cell-permeable small molecule, was commercially obtained. The LLL12 stock was dissolved in dimethyl sulfoxide (DMSO; Mallinckrodt Chemical Works, St Louis, MO, USA) at 0.01 M.

### Cell proliferation

The effect of the STAT3 inhibitor LLL12 on the proliferation of primary FISS cells was evaluated using the AlamarBlue Cell Viability Assay Reagent (G-Biosciences, St. Louis, MO, USA). Primary cells derived from feline normal soft tissue, which were not immunoreactive for STAT3, were also used in this assay to evaluate the inhibitory effect of LLL12 (data not shown). Cells at a concentration of 4.5 × 10^3^ per well were seeded onto 96 well plates in DMEM supplemented with 10% FBS and incubated at 37 °C overnight. The medium was then removed. After washing twice with PBS, a fresh culture medium (100 µL per well) at various concentrations (0.125, 0.25, 0.5, 1, and 2 µM) of LLL12 was added. The cells in the control group (0 µM) were treated with culture medium alone. The untreated control group was treated with the medium containing DMSO at a concentration equal to the diluent of 2 µM LLL12. The negative control was set up without cells to determine the background fluorescence that might be present. The alamarBlue dye in its oxidized form is blue in color and non-fluorescent. The growing cells cause a chemical reduction of the alamarBlue dye from non-fluorescent blue to fluorescent red. The continued growth of viable cells maintains a reducing environment (fluorescent, red), and the inhibition of growth maintains an oxidized environment (non-fluorescent, blue), which can be detected using a fluorescence or absorbance detector. The media containing the drug were removed, and 100 µL of 10% alamarBlue (G-Biosciences) in PBS was added to each well. After incubation for 3 h at 37 °C, the plate was removed, and the fluorescence was measured at excitation wavelengths of 570 and 595 nm. The percentage reduction of alamarBlue was calculated according to the equation directions provided by the manufacturer and PRISM software (PRISM 6, GraphPad Software, San Diego, CA, USA).

### Cell migration

Cells at a concentration of 4.5 × 10^4^ per well were seeded onto 24-well plates in DMEM supplemented with 1% FBS and incubated at 37 °C overnight. The cells were washed twice with the culture medium. The wounds were generated by scratching the confluent cell monolayer using a pipette tip. The remaining monolayered cells were then treated with LLL12 at concentrations of 2, 1, 0.5, 0.25, 0.125, and 0 µM or DMSO alone as the vehicle control. After washing with culture medium, the wounds were measured and photographed at 0 and 24 h after treatment. Cell migration for the closed distances was measured and compared to that of the control group. The distances were measured in micrometers using ImageJ (NIH, Bethesda, MD, USA). Fifty different measurements per treatment were performed to determine the average distance. The data presented are the averages of triplicate measurements.

### Statistical analysis

Comparisons were performed between the treated and untreated groups. Results are expressed as mean ± standard deviation. All statistical tests were performed using SAS version 9.4 (SAS Campus Drive, Cary, NC, USA). The Pearson correlation method was used to examine the correlations between the IHC score of STAT3, phospho-STAT3 (Tyr705), and tumor grading. Statistical significance was set at *P* < 0.05. For the in vitro studies, a one-way analysis of variance was used to analyze the results of the cell proliferation and wound healing assays. Between-group comparisons were performed using Duncan’s multiple range test. Statistical significance was set at *P* < 0.05.

## Supplementary Information

**Additional file 1.**

**Additional file 2.**

## Data Availability

The datasets generated and/or analysed during the current study are not publicly available dueto the privacy of the animals used in the study but are available from the corresponding author on reasonable request.

## Abbreviations

aFGF: acidic fibroblast growth factor
bFGF: basic fibroblast growth factor
α-SMA: alpha-smooth muscle actin
DMEM: Dulbecco’s modified Eagle’s medium
JAK: Janus kinase
STAT3: signal transducer and activator of transcription 3
FFPE: formalin-fixed and paraffin-embedded
FISS: Feline injection-site sarcomas
IHC: Immunohistochemistry
ICC: Immunocytochemistry
