# Supplementary material for: Investigating the role of signal transducer and activator of transcription 3 in feline injection site sarcoma
Source: BMC Vet Res. 2022 Jul 14;18:276. doi: 10.1186/s12917-022-03352-y (PMC9281114; doi:10.1186/s12917-022-03352-y)
Supplement: Supplementary file 1 — Additional file 1. [file 12917_2022_3352_MOESM1_ESM.pptx]

## Slide 1
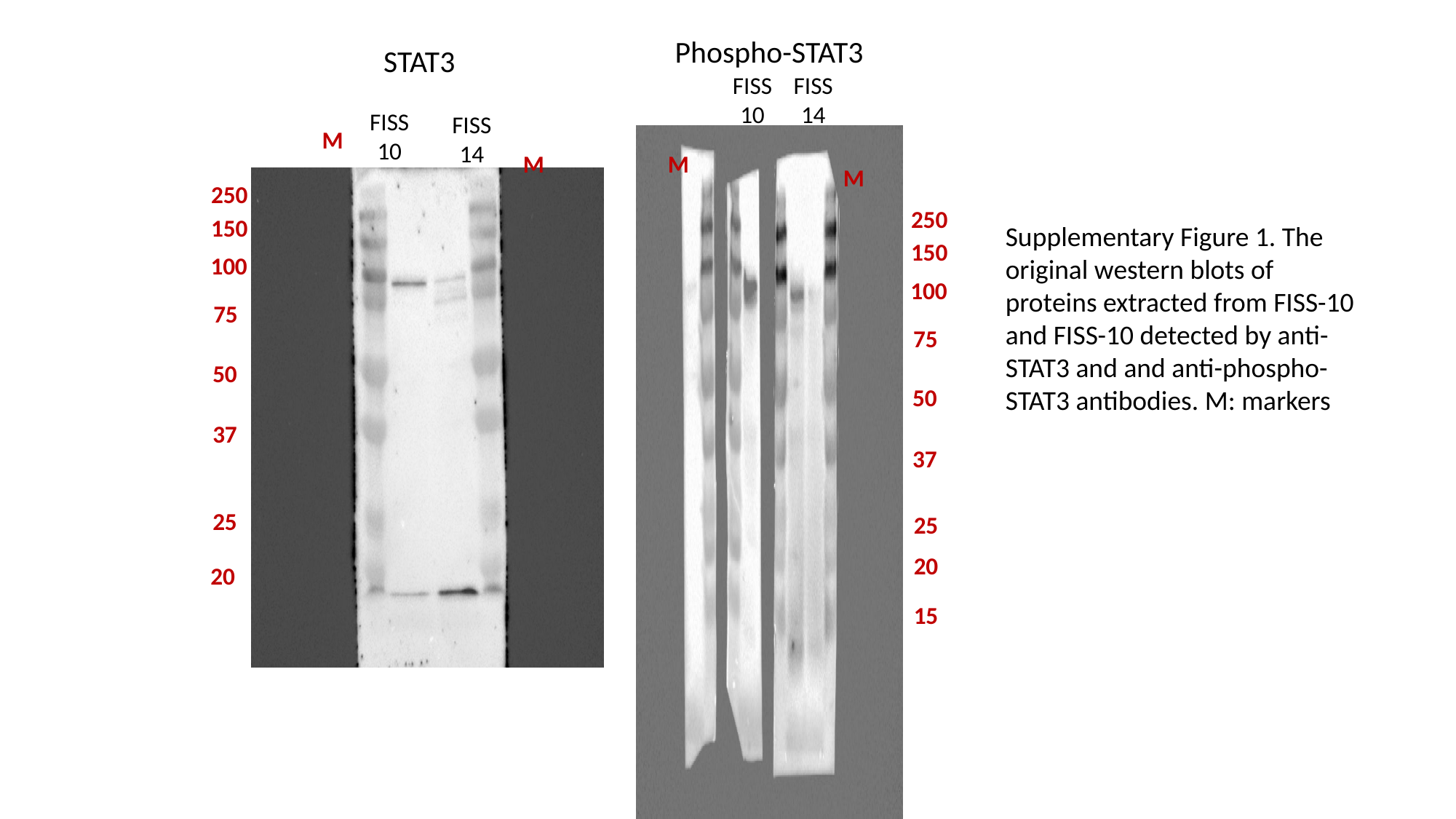

Phospho-STAT3
STAT3
FISS
10
FISS
14
FISS
10
FISS
14
M
M
M
M
250
250
150
Supplementary Figure 1. The original western blots of proteins extracted from FISS-10 and FISS-10 detected by anti-STAT3 and and anti-phospho-STAT3 antibodies. M: markers
150
100
100
75
75
50
50
37
37
25
25
20
20
15
