# Supplementary material for: Investigating the role of signal transducer and activator of transcription 3 in feline injection site sarcoma
Source: BMC Vet Res. 2022 Jul 14;18:276. doi: 10.1186/s12917-022-03352-y (PMC9281114; doi:10.1186/s12917-022-03352-y)
Supplement: Supplementary file 2 — Additional file 2. [file 12917_2022_3352_MOESM2_ESM.pptx]

## Slide 1
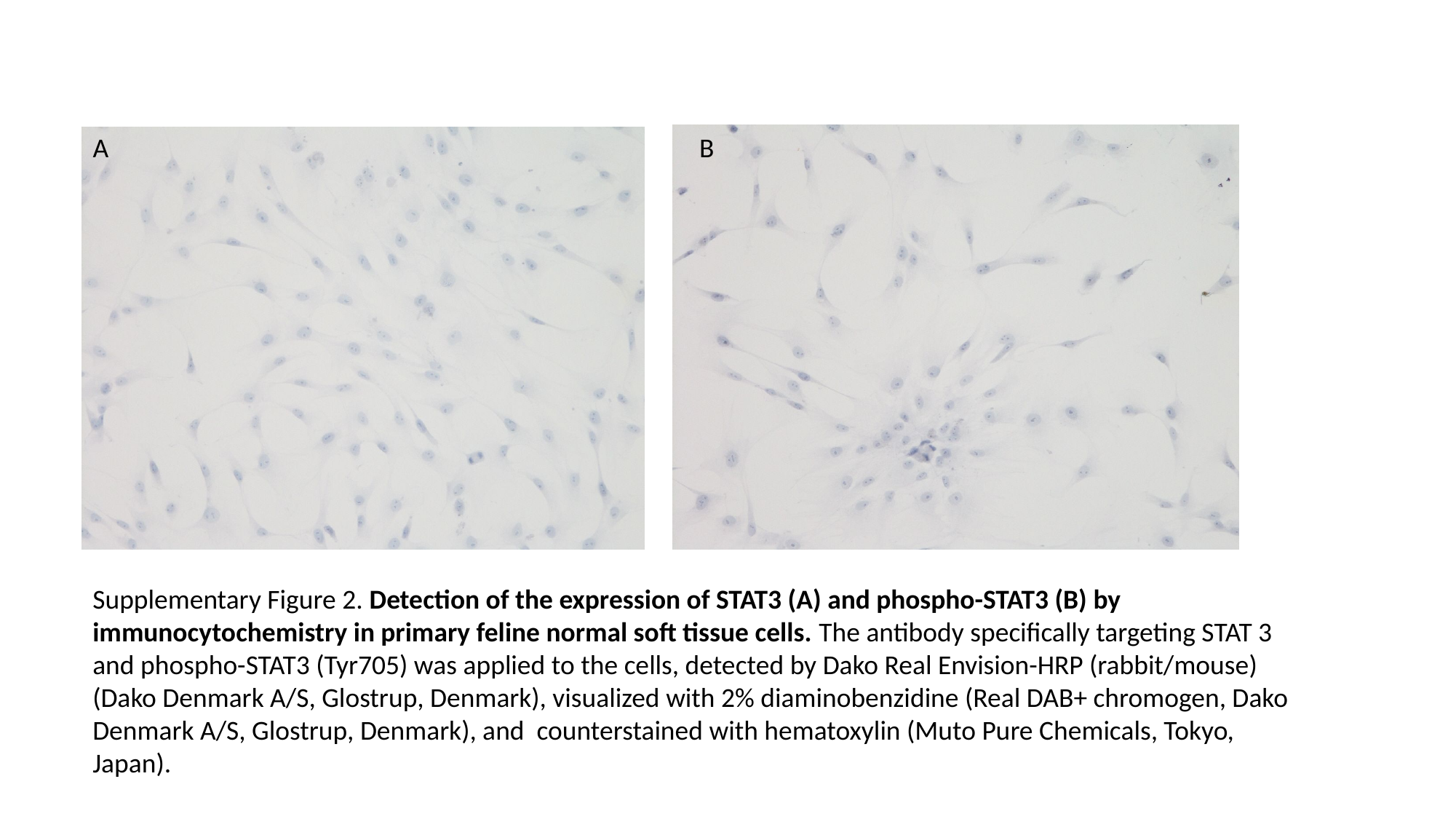

A
B
Supplementary Figure 2. Detection of the expression of STAT3 (A) and phospho-STAT3 (B) by immunocytochemistry in primary feline normal soft tissue cells. The antibody specifically targeting STAT 3 and phospho-STAT3 (Tyr705) was applied to the cells, detected by Dako Real Envision-HRP (rabbit/mouse) (Dako Denmark A/S, Glostrup, Denmark), visualized with 2% diaminobenzidine (Real DAB+ chromogen, Dako Denmark A/S, Glostrup, Denmark), and counterstained with hematoxylin (Muto Pure Chemicals, Tokyo, Japan).
